# Machine learning algorithms to predict risk of postoperative pneumonia in elderly with hip fracture

**DOI:** 10.1186/s13018-023-04049-0

**Published:** 2023-08-05

**Authors:** Jiale Guo, Qionghan He, Caiju Peng, Ru Dai, Wei Li, Zhichao Su, Yehai Li

**Affiliations:** 1 Department of Orthopedics, Chaohu Hospital of Anhui Medical University, Hefei, China; 2 Chaohu Hospital of Anhui Medical University, Hefei, China

**Keywords:** Machine learning, Hip fractures, Postoperative pneumonia, Predictive models, Risk factors

## Abstract

**Background:**

Hip fracture (HF) is one of the most common fractures in the elderly and is significantly associated with high mortality and unfavorable prognosis. Postoperative pneumonia (POP), the most common postoperative complication of HF, can seriously affect patient prognosis and increase the burden on the healthcare system. The aim of this study was to develop machine learning models for identifying elderly patients at high risk of pneumonia after hip fracture surgery.

**Methods:**

From May 2016 to November 2022, patients admitted to a single central hospital for HF served as the study population. We extracted data that could be collected within 24 h of patient admission. The dataset was divided into training and validation sets according to 70:30. Based on the screened risk factors, prediction models were developed using seven machine learning algorithms, namely CART, GBM, KNN, LR, NNet, RF, and XGBoost, and their performance was evaluated.

**Results:**

Eight hundred five patients were finally included in the analysis and 75 (9.3%) patients suffered from POP. Age, CI, COPD, WBC, HB, GLU, STB, GLOB, Ka^+^ which are used as features to build machine learning models. By evaluating the model's AUC value, accuracy, sensitivity, specificity, Kappa value, MCC value, Brier score value, calibration curve, and DCA curve, the model constructed by XGBoost algorithm has the best and near-perfect performance.

**Conclusion:**

The machine learning model we created is ideal for detecting elderly patients at high risk of POP after HF at an early stage.

**Supplementary Information:**

The online version contains supplementary material available at 10.1186/s13018-023-04049-0.

## Introduction

Hip fractures (HF) are devastating osteoporotic fractures, as they are closely associated with high morbidity, high mortality, and poor prognosis [1, 2]. HF, one of the most common fractures in older adults, accounts for more than 14% of fractures in older adults [3]. Although its incidence has declined in developed countries, the absolute incidence of HF is growing as population aging progresses worldwide [4–8]. The study indicates that the number of people with HF will increase to 6.3 million by 2050 [9]. Although different types of HF have different surgical options [10, 11], surgical treatment can significantly improve patient prognosis. The poor prognosis of HF is closely related to postoperative complications [12]. Effective perioperative management of patients with hip fractures can significantly reduce the number of postoperative complications [13, 14]. The most frequent postoperative complication in HF is postoperative pneumonia (POP), which increases mortality and length of hospital stay [15, 16]. In patients with POP, the risk of death increased to 3 times 43% at 30 days and to 2.4 times 71% at 1 year [15]. To improve patient prognosis, it is crucial to identify patients who are at high risk for developing postoperative pneumonia early and to take appropriate action. Machine learning (ML) algorithms are often used in the construction of clinical predictive models. As an important subfield of artificial intelligence, ML can learn from databases and have better predictive results for metrics than traditional linear models [17].

The aim of this study is to develop a machine learning algorithm prediction model to early identify patients at high risk of POP based on data collected at the time of patient admission to assist clinicians in decision-making, which can provide early intervention for high-risk patients and reduce the incidence of POP.

## Materials and methods

### Data collection

This study collected patients who were hospitalized for hip fractures at a university hospital from May 2016 to November 2022. Relevant medical record data information was extracted from the electronic medical record system. Inclusion criteria: (1). Patients admitted to the hospital for hip fracture; (2). The patient's age was not less than 60 years old. Exclusion criteria: (1). Not treated surgically; (2). Preoperative diagnosis of lung infection; (3). Multiple injuries; (4). Missing data information > 20%; (5). With acute cardiovascular or cerebrovascular disease, cancer, or other diseases that have a serious impact on the patient's prognosis; (6). Pathological fractures.

The diagnosis of POP is based on the Centers for Disease Control and Prevention's diagnostic criteria for POP [18]. In this study, the diagnosis of POP was based on the presence of the following events identifiable in the electronic medical record system in the time period after 24 h after surgery and before discharge: (1) new pulmonary infiltrative shadows, solid lesions, or cavity formation on imaging (X-ray or CT); (2) exclusion of other causes of fever (> 38 ^o^C), leukopenia (leukocyte count < 4 × 10^9^/L) or leukocytosis syndrome (leukocyte count > 12 × 10^9^/L), or for adults over 70 years of age with altered mental status excluding other recognized causes; (3) changes associated with increased respiratory secretions, coughing and sputum, dyspnea, pulmonary rales, or bronchial breath sounds were documented in the medical record system.

The general patient characteristics, prevalent geriatric chronic diseases, and prevalent laboratory test results available within 24 h of admission were among the variables we extracted. The specific items are detailed in Table 1. Since different testing reagents and modalities can produce different normal values for laboratory results, we converted all laboratory test results combined with clinical data into dichotomous variables based on whether they exceeded the upper limit or fell below the lower limit.

**Table 1:** Characteristics of patients in the training set

Variables	No-POP (*n* = 512)	POP (*n* = 51)	*p*
Female, *n* (%)	340 (66)	33 (65)	0.929
Age, Median (Q1, Q3)	78 (72, 84)	83 (79, 86.5)	< 0.001
Left, *n* (%)	269 (53)	30 (59)	0.477
Fracture. time, Median (Q1, Q3)	1 (1, 2)	1 (1, 2)	0.972
HBP, *n* (%)	259 (51)	23 (45)	0.548
CHD, *n* (%)	82 (16)	7 (14)	0.821
DM, *n* (%)	87 (17)	8 (16)	0.967
CI, *n* (%)	123 (24)	17 (33)	0.195
COPD, *n* (%)	40 (8)	11 (22)	0.003
FNF, *n* (%)	248 (48)	19 (37)	0.168
WBC [> 10 × 10^9^/L], *n* (%)	93 (18)	19 (37)	0.002
N [> 70%], *n* (%)	411 (80)	41 (80)	1
RBC [< lower limitation], *n* (%)	284 (55)	32 (63)	0.395
HB [< Lower Limitation, g/L], *n* (%)	311 (61)	36 (71)	0.219
PLT [< 100 × 10^9^/L], *n* (%)	75 (15)	6 (12)	0.726
GLU [> 6.1 mmol/L], *n* (%)	251 (49)	30 (59)	0.235
ALT [> 40u/L], *n* (%)	20 (4)	2 (4)	1
AST [> 40u/L], *n* (%)	26 (5)	4 (8)	0.338
STB [> 17.1umol/L], *n* (%)	247 (48)	32 (63)	0.067
DBIL [> 6.8umol/L], *n* (%)	224 (44)	29 (57)	0.099
IBIL [> 10.2umol/L], *n* (%)	269 (53)	32 (63)	0.213
ALB [< 35 g/L], *n* (%)	127 (25)	18 (35)	0.143
GLOB [> 35 g/L], *n* (%)	40 (8)	8 (16)	0.065
BUN [> 9.5 mmol/L], *n* (%)	105 (21)	11 (22)	1
Cr [> 97umol/L], *n* (%)	76 (15)	8 (16)	1
Ka^+^ [< 3.5 mmol/L], *n* (%)	138 (27)	8 (16)	0.113
Na^+^ [< 135 mmol/L], *n* (%)	25 (5)	3 (6)	0.733
Ca^+^ [< 2.18 mmol/L], *n* (%)	353 (69)	37 (73)	0.709

*Left* Fracture side, *Fracture.time* Time from injury to admission, *FNF* Femoral neck fracture (fracture type)

*HBP* High blood pressure, *CHD* Coronary heart disease, *DM* Diabetes mellitus, *CI* Cerebral infarction, *COPD* Chronic obstructive pulmonary disease, *WBC* White blood cell count, *N* Neutrophil ratio, *RBC* Red blood cell count, *HB* Hemoglobin, *PLT* Platelet count, *GLU* Blood glucose, *ALT* Alanine aminotransferase, *AST* Alanine aminotransferase, *STB* Sum bilirubin, *DBIL* Direct bilirubin, *IBIL* Indirect bilirubin, *ALB* Albumin, *GLOB* Globulin, *BUN* Blood urea nitrogen, *Cr* Creatinine, Ka^+^: potassium ion, Na^+^ Sodium ion, Ca^+^ Calcium ion

Two authors independently extracted the data, and a third author confirmed the veracity of the data. The study was approved by the hospital ethics review committee (number: KYXM-202302-005). An informed consent waiver was obtained because the study was retrospective and the personal information of the patients was withheld during the analysis. All procedures performed in this study were in accordance with the 1964 Declaration of Helsinki and its amendments.

### Statistical analysis

We use multiple interpolations to interpolate the missing data, which is done through the "mice" package in *R*. The median (interquartile range) was used to represent non-normally distributed continuous variables, and categorical variables were expressed as percentages. Continuous variables were analyzed using the Mann–Whitney *U*; categorical variables were analyzed using the chi-square test or Fisher test.

All patients included in the analysis were randomly divided into training and validation sets according to 70:30. To avoid the effect of multicollinearity among variables, we will use the Least Absolute Shrinkage and Selection Operator (LASSO) technique to perform screening of variables [19]. The screened variables were then subjected to correlation tests to clarify the presence of multicollinearity among the variables, and the correlation heatmap was drawn. The correlation coefficients were taken as [− 1,1], the larger the absolute value, the stronger the correlation, and greater than 0.4 indicated the existence of a significant correlation. The filtered variables are incorporated as final features in the model of the machine learning algorithm. Using Classification and Regression Tree (CART), Gradient Boosting Machine (GBM), k-Nearest Neighbors (KNN), Logistic Regression (LR), Neural Network (NNet), Random Forest (RF), and eXtreme Gradient Boosting (XGBoost), the seven machine learning algorithms to build prediction models. Ten times tenfold cross-validation resampling was used to ensure the stability and reproducibility of the model performance. The receiver operating characteristic (ROC) curve was used to evaluate the predictive performance of the model, and the higher the area under the curve (AUC) of the ROC, the better the model discrimination. Accuracy, sensitivity, specificity, Kappa value, and Matthews correlation coefficient (MCC) values were used as additional descriptions of the predictive ability of the model. The Kappa value is a metric to evaluate the consistency between the predicted and actual values of the model, and it takes the value of [− 1,1], the closer to 1, the better the consistency [20]. If it is > 0.75, the consistency is excellent, if it is between 0.40 and 0.75, the consistency is good, and if it is < 0.4, the consistency is poor. Due to the low incidence of positive events in this study, Matthews correlation coefficient (MCC) values provide a more balanced reflection of the model's predictive accuracy for a dataset with this imbalance problem. [21]. Its value is taken in [− 1,1], the closer to 1 the more perfect the prediction accuracy, above 0.5 is better, and greater than 0.7 indicates a high accuracy. The Brier Score is used to evaluate the calibration of the model and takes values in [0,1], the closer to 0 the better the calibration of the model, and less than 0.25 indicates that the calibration is acceptable [22]. Calibration curves were used as a complementary illustration of the calibration degree of the model. Decision curve analysis (DCA) is used to evaluate the clinical utility of the model in decision-making. Various evaluation metrics were combined to select the best machine learning algorithm prediction model. Shapely Additive exPlanations (SHAP) values were used to interpret the best machine learning models [23].

All statistical analyses, model construction and validation in this study were based on *R* software (version 4.1.3).

## Results

**Fig. 1:**
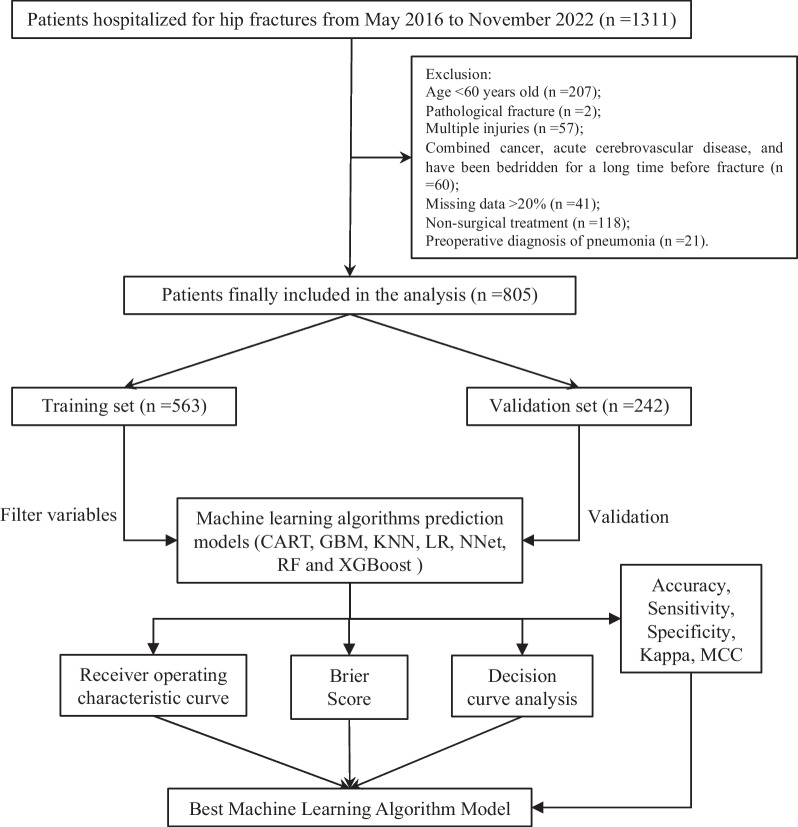
Flowchart of data screening and analysis

After screening based on inclusion and exclusion criteria, 805 patients were finally included in the study, and 75 (9.3%) patients suffered from POP, and the entire process of screening and analysis is shown in the flow chart (Fig. 1). The entire dataset was randomly divided 70:30 into a training set (*n* = 563) and a validation set (*n* = 242), and there were roughly no statistically significant differences between the two data (Additional file 1: S1.). We extracted 28 variables from each patient, and the patient characteristics in the training set are shown in Table 1.

**Fig. 2:**
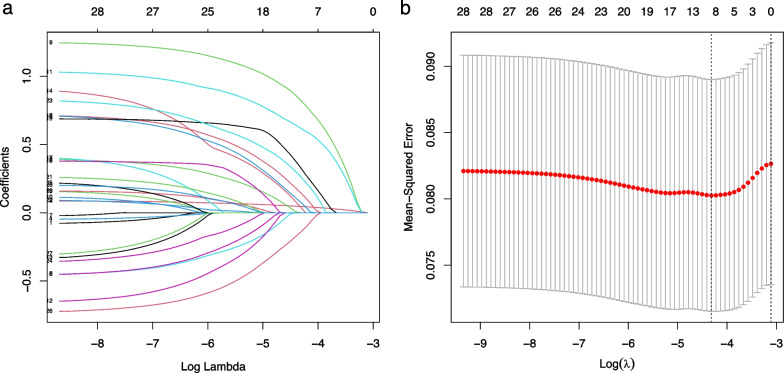
The potential risk factors were selected using the LASSO regression. **a** Trend graph of variance filter coefficients. Each color curve represents a trend in variance coefficient change. **b** Graph of cross-validation results. The vertical line on the left side represents *λ* min, and the vertical line on the right side represents *λ* 1se. *λ* min refers to the *λ* value corresponding to the minimum mean squared error (MSE) among all *λ* values; *λ* 1se refers to the *λ* value corresponding to the simplest and best model obtained after cross-validation within a square difference range of *λ* min

**Fig. 3:**
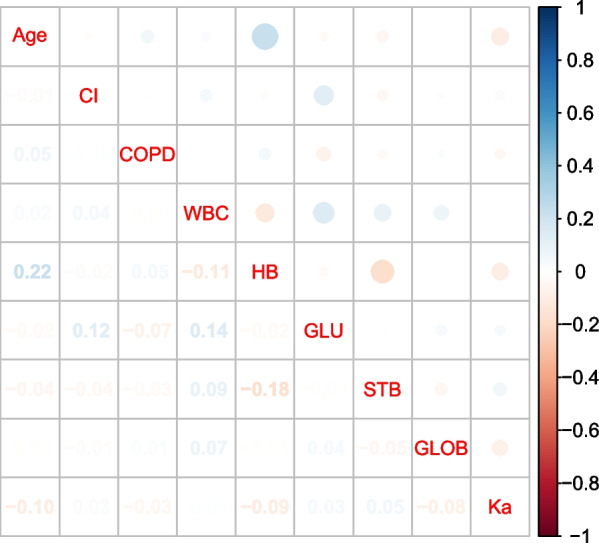
Heatmap of correlation analysis between variables

To avoid multicollinearity among the variables included in the model, LASSO regression was used to screen the features included in the model, and the results showed that when the lambda value was chosen as lambda.min (0.01331355), a total of nine features with nonzero coefficients were screened (Fig. 2), namely Age, CI, COPD, WBC, HB, GLU, STB GLOB, and Ka. Further correlation analysis was performed to analyze the correlations among these nine variables and a correlation heatmap was drawn (Fig. 3). The correlations of all variables were less than 0.4, indicating that there were no significant correlations among the screened variables. The screened variables were used as features to construct prediction models using seven machine learning algorithms (CART, GBM, KNN, LR, NNet, RF, XGBoost).

**Fig. 4:**
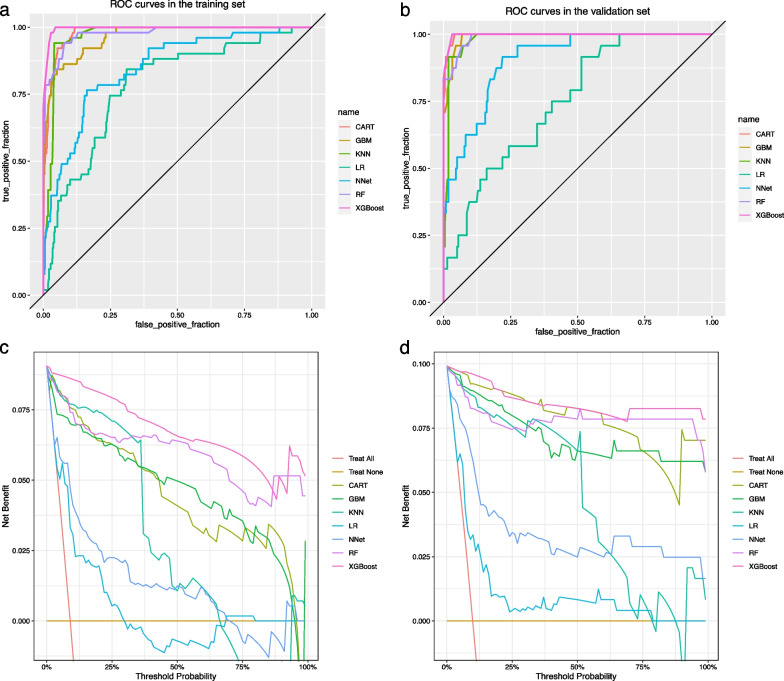
ROC curves and DCA curves for each model in the training and validation sets. **a** ROC curves in the training set. **b** ROC curves in the validation set. **c** DCA curves in the training set. **d** DCA curves in the validation set

**Fig. 5:**
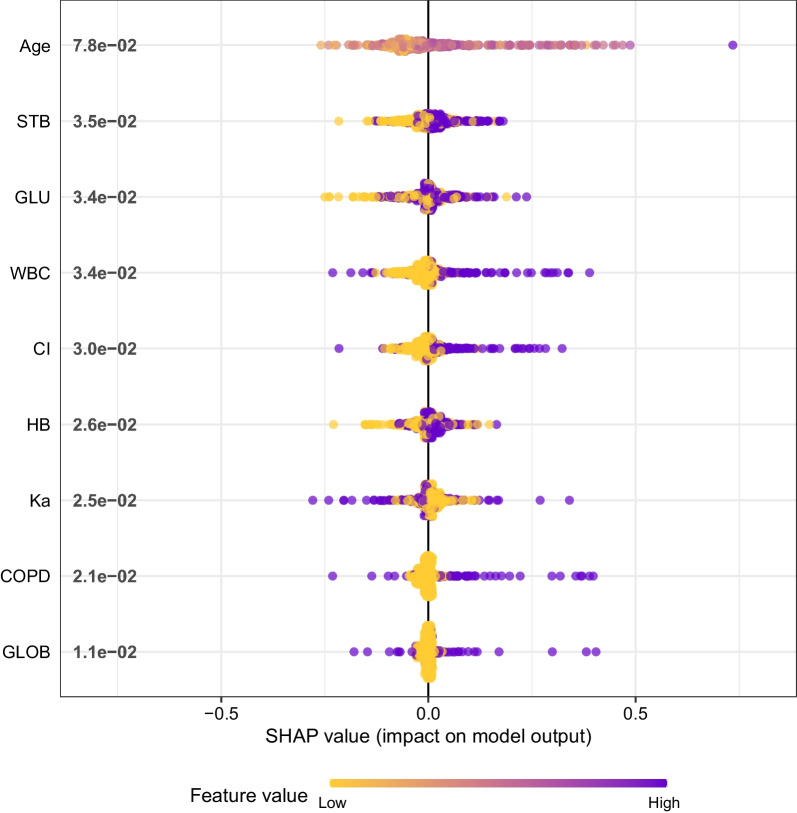
Summary plot of SHAP values for the model constructed by XGBoost algorithm. The vertical coordinates show the importance of the features, sorted by the importance of the variables in descending order, with the upper variables being more important to the model. For the horizontal position "SHAP value" shows whether the impact of the value is associated with a higher or lower prediction. The color of each SHAP value point indicates whether the observed value is higher (purple) or lower (yellow)

The performance of the models constructed by each algorithm was determined by resampling with ten times tenfold cross validation. The AUC values were calculated based on the ROC curves.The AUC values (95% confidence interval) of CART, GBM, KNN, LR, NNet, RF, and XGBoost algorithms in the training set (Fig. 4a) were 0.981 (0.971, 0.991), 0.965 (0.945, 0.985), 0.969 (0.956, 0.983) 0.983), 0.784 (0.72, 0.849), 0.849 (0.794, 0.904), 0.978 (0.96, 0.996), and 0.996 (0.992, 0.999); the AUC values (95% confidence interval) in the validation set (Fig. 4b) were 0.997 (0.993, 1), 0.991 ( 0.982, 1), 0.983 (0.968, 0.997), 0.75 (0.658, 0.841), 0.907 (0.855, 0.958), 0.99 (0.979, 1), and 0.998 (0.994, 1), respectively (Table 2). The ROC curves of the models constructed by each algorithm are shown in Additional file 1: S2-S15. Accuracy, sensitivity, specificity, Kappa value, and MCC value as additional descriptions of the predictive ability of the models are shown in Table 2. For datasets with unbalanced distribution of results, the MCC value reflects the actual predictive ability of the model better than the AUC value. The MCC values show that only the models constructed by KNN and XGBoost algorithms have good accuracy. The Brier scores of CART, GBM, KNN, LR, NNet, RF, and XGBoost algorithms in the training set are: 0.038, 0.038, 0.047, 0.075, 0.065, 0.041, and 0.017, respectively; in the validation set are: 0.023, 0.029, 0.051, 0.081, and 0.058, 0.042, 0.016, respectively (Table 2). The Brier Score of each model is less than 0.25, indicating that the calibration degree of each model is fine. The calibration curves, as a supplement to the calibration degree, are shown in Additional file 1: S16–S29 for the models constructed by each algorithm. The DCA curves show that in both the training set (Fig. 4c) and the validation set (Fig. 4d), the models achieve higher net returns than the "all-intervention" or "no-intervention" strategies over a wide range of thresholds. The DCA curves of the models constructed by each algorithm are shown in S30-S43. Combining the results of each model performance evaluation, the model constructed by the XGBoost algorithm shows the best performance. We further plotted a summary plot of SHAP values to interpret the XGBoost model results (Fig. 5). For each feature, a point corresponds to a patient, and the position of the point on the x-axis (i.e., the actual SHAP value) indicates the effect of the feature on the model output for that particular patient. The vertical coordinates show the importance of the features, with Age, STB, and GLU being the top three variables important to the model.

**Table 2:** Evaluation metrics of the models constructed by each algorithm

	AUC	ACC	SEN	SPE	Kappa	Brier score	MCC
*Train*							
CART	0.981	0.893	1	0.883	0.577	0.038	0.637
GBM	0.965	0.94	0.843	0.949	0.684	0.038	0.694
KNN	0.969	0.957	0.941	0.959	0.777	0.047	0.788
LR	0.784	0.703	0.843	0.689	0.228	0.075	0.319
NNet	0.849	0.831	0.765	0.838	0.37	0.065	0.42
RF	0.978	0.924	0.941	0.922	0.651	0.041	0.682
XGBoost	0.996	0.959	1	0.955	0.794	0.017	0.881
*Valid*							
CART	0.997	0.963	1	0.959	0.822	0.023	0.835
GBM	0.991	0.938	1	0.931	0.729	0.029	0.757
KNN	0.983	0.975	0.917	0.982	0.866	0.051	0.867
LR	0.75	0.529	0.917	0.486	0.133	0.081	0.242
NNet	0.907	0.793	0.917	0.78	0.376	0.058	0.459
RF	0.99	0.905	1	0.894	0.627	0.042	0.676
XGBoost	0.998	0.971	1	0.968	0.857	0.016	0.866

*Train* Training set

*Valid* Validation set

*AUC* Area under the curve

*ACC* Accuracy

*SEN* Sensitivity

*SPE* Specificity

*MCC* Matthews correlation coefficient

## Discussion

Predictive tools are becoming increasingly common in clinical practice, and these tools are often developed based on data sets to be used for clinical prognosis and diagnosis prediction [24–27]. Traditional linear regression and supervised machine learning algorithms are commonly used to construct models. In this study, predictive models of machine learning algorithms were constructed to predict the risk of pneumonia after hip fracture surgery in elderly people based on the early admission data of patients. This study constructed models based on seven commonly used machine learning algorithms, and the model based on the XGBoost algorithm performed best in terms of model performance. The model constructed in this study can identify patients at high risk of postoperative pneumonia after hip fracture at an early stage of hospital admission. Early intervention in high-risk patients can prevent postoperative pneumonia to a certain extent, improve patient prognosis and reduce the medical burden.

It has been reported that China's aging population over 60 years of age has reached 249 million as of 2018, and this population is expected to exceed 450 million by 2050 [28]. As the population ages, the number of hip fractures will continue to increase. As the "last fracture of life", hip fractures are essential to receive surgical treatment. Surgical treatment can significantly reduce the 1-year mortality rate of patients [29]. POP is the most common postoperative complication of hip fracture in the elderly, with an incidence of 4.9% to 15.2% [30–32]. POP is strongly associated with many short-term and long-term prognoses, including prolonged hospital stays, ICU admissions, readmission rates, and mortality [33]. Therefore, the ability to reduce the incidence of POP by intervening earlier would provide many benefits to the patient, the patient's family, and the social health care system. A number of variables have been found to be risk factors for POP after hip fracture, such as preoperative hypoproteinemia, COPD, CI, age, male, anemia, and diabetes mellitus [30, 32, 34]. In addition, surgery-related factors, such as time from injury to surgery, duration of surgery, and type of anesthesia, have also been shown to be high-risk factors for POP [32, 35]. The results of our analysis were similar to theirs. The variables we included in the models have been shown to be associated with postoperative pneumonia in many studies [36, 37].

A nomogram has been constructed by Zhang et al. [38] and Xiang et al. [35] for predicting pneumonia after hip fracture surgery in the elderly. Both of their nomograms have good AUC values (0.84 and 0.905, respectively), however simply reporting AUC values for data with an unbalanced distribution of dichotomous results is not sufficient. Even more important is the ability of the model to predict positive events. Our model addresses this issue well by reporting MCC values. Furthermore, the variables they included in their analysis all included variables related to surgery, such as time of surgery, time from injury to surgery, and type of surgery. This prevents the identification of high-risk patients early in their admission and timely intervention. However, the variables included in the model for this study could be collected quickly and easily in all regions, which facilitated the use of the tool. More importantly, we are the first study to apply machine learning algorithms to predict pneumonia after hip fracture surgery. Based on the current exploration in the field of artificial intelligence, it is necessary to apply common machine learning algorithms to this field for experimentation.

However, there are still some limitations in this study. (1). This is a retrospective study, and retrospective bias and selection bias of the data are difficult to avoid. (2). All data were obtained from a single center, and there was some bias in the selection of the population, so there may be some limitations in the application of the model to populations in other regions. (3). The amount of data included in the analysis was small. Although the sample size of this study met the basic requirements for constructing the model, the sample size was still not large enough [39, 40]. In particular, a sufficiently large sample size is required to construct models for machine learning algorithms. Based on these, we need a large sample size of multicenter prospective studies for further validation of this study.

## Summary

In this study, seven machine learning algorithms, CART, GBM, KNN, LR, NNet, RF, and XGBoost, were used to construct models to predict postoperative pneumonia in elderly people with hip fracture. The model based on XGBoost algorithm has excellent performance and can be used to clinically assist physicians in decision making to identify high-risk patients early in hospital admission and intervene earlier.

### Supplementary Information

**Additional file 1:** Characterization of the training and validation sets and evaluation plots of the performance of each model.

## Data Availability

The data that support the findings of this study are available from the corresponding author upon reasonable request.

## References

[1] Lawrence VA, Hilsenbeck SG, Noveck H, Poses RM, Carson JL (2002). Medical complications and outcomes after hip fracture repair. Arch Intern Med.

[2] Chen YP, Kuo YJ, Hung SW, Wen TW, Chien PC, Chiang MH (2021). Loss of skeletal muscle mass can be predicted by sarcopenia and reflects poor functional recovery at one year after surgery for geriatric hip fractures. Injury.

[3] Burge R, Dawson-Hughes B, Solomon DH, Wong JB, King A, Tosteson A (2007). Incidence and economic burden of osteoporosis-related fractures in the United States, 2005–2025. J Bone Miner Res.

[4] Dimai HP, Reichardt B, Zitt E, Concin H, Malle O, Fahrleitner-Pammer A (2022). Thirty years of hip fracture incidence in Austria: is the worst over?. Osteoporos Int.

[5] Kannus P, Niemi S, Parkkari J, Sievänen H (2018). Continuously declining incidence of hip fracture in Finland: analysis of nationwide database in 1970–2016. Arch Gerontol Geriatr Jul-Aug.

[6] Cauley JA, Chalhoub D, Kassem AM, Fuleihan GH (2014). Geographic and ethnic disparities in osteoporotic fractures. Nat Rev Endocrinol.

[7] Nordström P, Bergman J, Ballin M, Nordström A (2022). Trends in hip fracture incidence, length of hospital stay, and 30-day mortality in Sweden from 1998–2017: a nationwide cohort study. Calcif Tissue Int.

[8] Wu AM, Bisignano C, James SL, Abady GG, Abedi A, Abu-Gharbieh E, Alhassan RK, Alipour V, Arabloo J, Asaad M, Asmare WN (2021). Global, regional, and national burden of bone fractures in 204 countries and territories, 1990–2019: a systematic analysis from the global burden of disease study 2019. Lancet Healthy Longev.

[9] Cooper C, Campion G, Melton LJ (1992). Hip fractures in the elderly: a world-wide projection. Osteoporos Int.

[10] Marsillo E, Pintore A, Asparago G, Oliva F, Maffulli N (2022). Cephalomedullary nailing for reverse oblique intertrochanteric fractures 31A3 (AO/OTA). Orthop Rev.

[11] Gargano G, Poeta N, Oliva F, Migliorini F, Maffulli N (2021). Zimmer natural nail and ELOS nails in pertrochanteric fractures. J Orthop Surg Res.

[12] Wang X, Zhao BJ, Su Y (2017). Can we predict postoperative complications in elderly Chinese patients with hip fractures using the surgical risk calculator?. Clin Interv Aging.

[13] Maffulli N, Aicale R (2022). Proximal femoral fractures in the elderly: a few things to know, and some to forget. Medicina.

[14] Quaranta M, Miranda L, Oliva F, Migliorini F, Pezzuti G, Maffulli N (2021). Haemoglobin and transfusions in elderly patients with hip fractures: the effect of a dedicated orthogeriatrician. J Orthop Surg Res.

[15] Roche JJ, Wenn RT, Sahota O, Moran CG (2005). Effect of comorbidities and postoperative complications on mortality after hip fracture in elderly people: prospective observational cohort study. Bmj.

[16] Fernandez-Bustamante A, Frendl G, Sprung J, Kor DJ, Subramaniam B, Martinez Ruiz R (2017). Postoperative pulmonary complications, early mortality, and hospital stay following noncardiothoracic surgery: a multicenter study by the perioperative research network investigators. JAMA Surg.

[17] Deo RC (2015). Machine learning in medicine. Circulation.

[18] Abbott TE, Fowler AJ, Pelosi P, De Abreu MG, Møller AM, Canet J, Creagh-Brown B, Mythen M, Gin T, Lalu MM, Futier E (2018). A systematic review and consensus definitions for standardised end-points in perioperative medicine: pulmonary complications. Br J Anaesth.

[19] Tibshirani R (1996). Regression shrinkage and selection via the Lasso. J R Stat Soc Ser B: Stat Methodol.

[20] McHugh ML (2012). Interrater reliability: the kappa statistic. Biochemia Medica.

[21] Boughorbel S, Jarray F, El-Anbari M (2017). Optimal classifier for imbalanced data using matthews correlation coefficient metric article. Plos One.

[22] Hilden J, Habbema JD, Bjerregaard B (1978). The measurement of performance in probabilistic diagnosis. III. Methods based on continuous functions of the diagnostic probabilities. Methods Inf Med.

[23] Lundberg SM, Lee SI. A unified approach to interpreting model predictions. In: 31st Annual conference on neural information processing systems (NIPS), Long Beach, CA; 2017.

[24] Lex JR, Di Michele J, Koucheki R, Pincus D, Whyne C, Ravi B (2023). Artificial intelligence for hip fracture detection and outcome prediction: a systematic review and meta-analysis. JAMA Netw Open.

[25] Zhou Y, Gould D, Choong P, Dowsey M, Schilling C (2022). Implementing predictive tools in surgery: a narrative review in the context of orthopaedic surgery. ANZ J Surg.

[26] Bernardini B, Baratto L, Pizzi C, Biggeri A, Cerina G, Colantonio V (2023). A multicenter prospective study validated a nomogram to predict individual risk of dependence in ambulation after rehabilitation. J Clin Epidemiol.

[27] Wang M, Chen X, Cui W, Wang X, Hu N, Tang H (2022). A computed tomography-based radiomics nomogram for predicting osteoporotic vertebral fractures: a longitudinal study. J Clin Endocrinol Metab.

[28] Zhang Q, Wu Y, Han T, Liu E (2019). Changes in cognitive function and risk factors for cognitive impairment of the elderly in China: 2005–2014. Int J Environ Res Pub Health.

[29] Chlebeck JD, Birch CE, Blankstein M, Kristiansen T, Bartlett CS, Schottel PC (2019). Nonoperative geriatric hip fracture treatment is associated with increased mortality: a matched cohort study. J Orthop Trauma.

[30] Lv H, Yin P, Long A, Gao Y, Zhao Z, Li J (2016). Clinical characteristics and risk factors of postoperative pneumonia after hip fracture surgery: a prospective cohort study. Osteoporos Int.

[31] Salarbaks AM, Lindeboom R, Nijmeijer W (2020). Pneumonia in hospitalized elderly hip fracture patients: the effects on length of hospital-stay, in-hospital and thirty-day mortality and a search for potential predictors. Injury.

[32] Wang Y, Li X, Ji Y, Tian H, Liang X, Li N (2019). Preoperative serum albumin level as a predictor of postoperative pneumonia after femoral neck fracture surgery in a geriatric population. Clin Interv Aging.

[33] Gao YC, Zhang YW, Shi L, Gao W, Li YJ, Chen H (2023). What are risk factors of postoperative pneumonia in geriatric individuals after hip fracture surgery: a systematic review and meta-analysis. Orthop Surg.

[34] Tian Y, Zhu Y, Zhang K, Tian M, Qin S, Li X (2022). Incidence and risk factors for postoperative pneumonia following surgically treated hip fracture in geriatric patients: a retrospective cohort study. J Orthop Surg Res.

[35] Xiang G, Dong X, Xu T, Feng Y, He Z, Ke C (2020). A nomogram for prediction of postoperative pneumonia risk in elderly hip fracture patients. Risk Manag Healthc Policy.

[36] Chen C, Yang D, Gao S, Zhang Y, Chen L, Wang B (2021). Development and performance assessment of novel machine learning models to predict pneumonia after liver transplantation. Respir Res.

[37] Yuan K, Li R, Zhao Y, Wang K, Lin F, Lu J (2022). Pre-operative predictors for post-operative pneumonia in aneurysmal subarachnoid hemorrhage after surgical clipping and endovascular coiling: a single-center retrospective study. Front Neurol.

[38] Zhang X, Shen ZL, Duan XZ, Zhou QR, Fan JF, Shen J (2022). Postoperative pneumonia in geriatric patients with a hip fracture: incidence, risk factors and a predictive nomogram. Geriatr Orthop Surg Rehabil.

[39] Riley RD, Ensor J, Snell KIE, Harrell FE, Martin GP, Reitsma JB (2020). Calculating the sample size required for developing a clinical prediction model. Bmj.

[40] Peduzzi P, Concato J, Feinstein AR, Holford TR (1995). Importance of events per independent variable in proportional hazards regression analysis. II. Accuracy and precision of regression estimates. J Clin Epidemiol.

