# Supplementary material for: Machine learning algorithms to predict risk of postoperative pneumonia in elderly with hip fracture
Source: J Orthop Surg Res. 2023 Aug 5;18:571. doi: 10.1186/s13018-023-04049-0 (PMC10403839; doi:10.1186/s13018-023-04049-0)
Supplement: Supplementary file 1 — Additional file 1: Characterization of the training and validation sets and evaluation plots of the performance of each model. [file 13018_2023_4049_MOESM1_ESM.docx]

S1. Characteristics of the training and validation sets

| Variables | Total (n = 805) | Train (n = 563) | Valid (n = 242) | p |
| --- | --- | --- | --- | --- |
| Female, n (%) | 528 (66) | 373 (66) | 155 (64) | 0.601 |
| Age, Median (Q1, Q3) | 78 (72, 85) | 79 (72, 85) | 78 (72, 84) | 0.546 |
| Left, n (%) | 411 (51) | 299 (53) | 112 (46) | 0.089 |
| Fracture. time, Median (Q1, Q3) | 1 (1, 2) | 1 (1, 2) | 1 (1, 2) | 0.313 |
| HBP, n (%) | 392 (49) | 282 (50) | 110 (45) | 0.259 |
| CHD, n (%) | 133 (17) | 89 (16) | 44 (18) | 0.467 |
| DM, n (%) | 134 (17) | 95 (17) | 39 (16) | 0.872 |
| CI, n (%) | 203 (25) | 140 (25) | 63 (26) | 0.794 |
| COPD, n (%) | 78 (10) | 51 (9) | 27 (11) | 0.428 |
| FNF, n (%) | 390 (48) | 267 (47) | 123 (51) | 0.419 |
| WBC [>10×10^9^/L], n (%) | 164 (20) | 112 (20) | 52 (21) | 0.675 |
| N [>70%], n (%) | 657 (82) | 452 (80) | 205 (85) | 0.165 |
| RBC [< lower limitation], n (%) | 445 (55) | 316 (56) | 129 (53) | 0.509 |
| HB [<Lower Limitation, g/L], n (%) | 494 (61) | 347 (62) | 147 (61) | 0.874 |
| PLT [<100×10^9^/L], n (%) | 120 (15) | 81 (14) | 39 (16) | 0.601 |
| GLU [>6.1mmol/L], n (%) | 392 (49) | 281 (50) | 111 (46) | 0.329 |
| ALT [>40u/L], n (%) | 30 (4) | 22 (4) | 8 (3) | 0.833 |
| AST [>40u/L], n (%) | 40 (5) | 30 (5) | 10 (4) | 0.59 |
| STB [>17.1umol/L], n (%) | 399 (50) | 279 (50) | 120 (50) | 1 |
| DBIL [>6.8umol/L], n (%) | 368 (46) | 253 (45) | 115 (48) | 0.55 |
| IBIL [>10.2umol/L], n (%) | 441 (55) | 301 (53) | 140 (58) | 0.285 |
| ALB [<35g/L], n (%) | 233 (29) | 145 (26) | 88 (36) | 0.003 |
| GLOB [>35g/L], n (%) | 60 (7) | 48 (9) | 12 (5) | 0.105 |
| BUN [>9.5mmol/L], n (%) | 169 (21) | 116 (21) | 53 (22) | 0.749 |
| Cr [>97umol/L], n (%) | 124 (15) | 84 (15) | 40 (17) | 0.636 |
| Ka^+^ [<3.5mmol/L], n (%) | 224 (28) | 146 (26) | 78 (32) | 0.081 |
| Na^+^ [<135mmol/L], n (%) | 43 (5) | 28 (5) | 15 (6) | 0.591 |
| Ca^+^ [<2.18mmol/L], n (%) | 554 (69) | 390 (69) | 164 (68) | 0.734 |
| POP, n (%) | 75 (9) | 51 (9) | 24 (10) | 0.801 |

S2. ROC curve of CART algorithm in the training set

S3. ROC curve of GBM algorithm in the training set

S4. ROC curve of KNN algorithm in the training set

S5. ROC curve of LR algorithm in the training set

S6. ROC curve of NNet algorithm in the training set

S7. ROC curve of RF algorithm in the training set

S8. ROC curve of XGBoost algorithm in the training set

S9. ROC curve of CART algorithm in the validation set

S10. ROC curve of GBM algorithm in the validation set

S11. ROC curve of KNN algorithm in the validation set

S12. ROC curve of LR algorithm in the validation set

S13. ROC curve of NNet algorithm in the validation set

S14. ROC curve of RF algorithm in the validation set

S15. ROC curve of XGBoost algorithm in the validation set

S16. Calibration curve of CART algorithm in the training set

S17. Calibration curve of GBM algorithm in the training set

S18. Calibration curve of KNN algorithm in the training set

S19. Calibration curve of LR algorithm in the training set

S20. Calibration curve of NNet algorithm in the training set

S21. Calibration curve of RF algorithm in the training set

S22. Calibration curve of XGBoost algorithm in the training set

S23. Calibration curve of CART algorithm in the validation set

S24. Calibration curve of GBM algorithm in the validation set

S25. Calibration curve of KNN algorithm in the validation set

S26. Calibration curve of LR algorithm in the validation set

S27. Calibration curve of NNet algorithm in the validation set

S28. Calibration curve of RF algorithm in the validation set

S29. Calibration curve of XGBoost algorithm in the validation set

S30. DCA curve of CART algorithm in the training set

S31. DCA curve of GBM algorithm in the training set

S32. DCA curve of KNN algorithm in the training set

S33. DCA curve of LR algorithm in the training set

S34. DCA curve of NNet algorithm in the training set

S35. DCA curve of RF algorithm in the training set

S36. DCA curve of XGBoost algorithm in the training set

S37. DCA curve of CART algorithm in the validation set

S38. DCA curve of GBM algorithm in the validation set

S39. DCA curve of KNN algorithm in the validation set

S40. DCA curve of LR algorithm in the validation set

S41. DCA curve of NNet algorithm in the validation set

S42. DCA curve of RF algorithm in the validation set

S43. DCA curve of XGBoost algorithm in the validation set
